# Traumatic Tonsillar Hemorrhage during Hemophilia A Treatment with Emicizumab

**DOI:** 10.3390/hematolrep14020016

**Published:** 2022-03-30

**Authors:** Fumiya Inoue, Kazuki Terada, Kazuki Furudate, Yasushi Noguchi, Shunji Igarashi

**Affiliations:** 1 Department of Pediatrics, Narita Red Cross Hospital, Narita City 286-8523, Japan; fumiyainoue2380@gmail.com (F.I.); dattekodomo@sweet.ocn.ne.jp (K.F.); nogunari@naritasekijyuji.jp (Y.N.); shun2@cd5.so-net.ne.jp (S.I.); 2 Department of Pediatric Hematology and Oncology, Narita Red Cross Hospital, Narita City 286-8523, Japan

**Keywords:** emicizumab, hemophilia A, hemostasis, tonsil, activated partial thromboplastin time

## Abstract

Reports on the treatment of bleeding associated with emicizumab administration are scarce. Herein, we report the case of an eight-year-old boy with moderate hemophilia A with an inhibitor who experienced tonsillar hemorrhage while undergoing emicizumab treatment. He visited our hospital for postprandial bloody vomiting. The activated partial thromboplastin time was 20.8 s; only a small amount of hemorrhage was observed in the retropharyngeal space, and tranexamic acid was administered. He experienced hematemesis on Day 2 of hospitalization, and fiberoptic laryngoscopy confirmed hemorrhage from the posterior tonsil. Varicose vessels were observed at the soft palate, and considering thrombosis, an emergency cauterization was performed instead of bypass therapy. In small children, observing the tonsils is difficult, and the coagulation ability of the patient with hemophilia A is inferior to that of healthy people, even under emicizumab administration. Thus, active hemorrhage assessment and appropriate hemostatic control are necessary.

## 1. Introduction

Regular replacement therapy, which prevents hemorrhage by replacing the coagulation factor VIII, is generally common in hemophilia A. Although the frequency of replacement decreases with the development of extended half-life factor VIII, some cases are difficult to treat due to the requirement of intravenous injection and the presence of factor VIII inhibitors; this remains a problem in administering the therapy [1]. However, in recent years, emicizumab, a monoclonal antibody that promotes coagulation reactions by bridging factors IXa and X, was introduced. Compared with the regular replacement therapy with coagulation factors, emicizumab provides more effective hemostatic effect and, due to administration through subcutaneous injection, a longer dose interval [2,3,4,5]; emicizumab exhibits reduced invasiveness. However, reports on the treatment of unexpected bleeding, as seen in trauma, are scarce, and to the best of our knowledge, there have been no previous reports on tonsillar hemorrhage that is particularly associated with emicizumab administration. Herein, we present a case report of a patient who experienced traumatic tonsillar hemorrhage while undergoing emicizumab treatment and for whom hemostatic control was difficult.

## 2. Case Report

**Figure 1:**
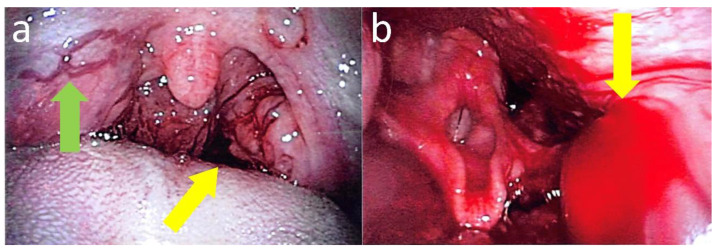
(**a**) Pharyngeal fiberoptic findings at the time of presentation. The yellow arrow indicates slight bleeding of the pharynx. The green arrow indicates varicose veins of the right soft palate. (**b**) Laryngeal fiberoptic findings during hematemesis. The yellow arrow indicates active bleeding from the posterior part of the left tonsil.

The patient was an eight-year-old boy. In terms of family history, he belonged to a fatherless family, paternal details were unclear, and carrier detection in the maternal family line was not performed. At infancy, there was an episode of hemostasis difficulty for gingival hemorrhage. Later, there were no episodes of apparent hemorrhage, but subcutaneous bleeding occurred repeatedly at six years of age; hence, he visited a local clinic. Swelling at the blood collection site during the blood test led to the diagnosis of moderate hemophilia A (Factor VIII activity 1.2% by chromogenic assay). Regular replacement of coagulation factor VIII was started; however, the patient was confirmed as inhibitor-positive one month later, and the titer was high at 31BU/mL. Afterward, regular replacement was not performed, and hemorrhage was handled with the replacement of coagulation factor VII. Four months later, the inhibitor titer was high, which increased up to 301 BU/mL. Two years and eleven months after the start of regular replacement therapy, the inhibitor titer decreased to 5 BU/mL but did not disappear. Immune tolerance therapy was also considered, but various reasons—such as the moderate severity level, difficulty in securing vascular access due to obesity, and repetition of traumatic subcutaneous bleeding in joints every one to two months—led to the induction of emicizumab. Two months before hospitalization, emicizumab induction was started with weekly administration of 3 mg/kg for four weeks, followed by biweekly administration of 3 mg/kg, and the course was satisfactory with no hemorrhage. The last emicizumab replacement was performed 12 days before hospitalization. The patient consumed sugar-coated sweet potato chips at night and was presented to the emergency room of our institution because of repeated hematemesis in the subsequent morning. He was hospitalized for detailed examination and treatment. The patient had a height of 144 cm (+3.5 standard deviation or SD), weight 50.2 kg (+4.8 SD), body temperature 36.7 °C, heart rate 110 bpm, blood pressure 126/82 mmHg, respiratory rate 20 breaths/min, and SpO_2_ 99% (room air). Bilateral second-degree tonsillar enlargement, a small amount of accumulated blood in the oral cavity, and venous distention in the right soft palate were observed. Hemoglobin concentration (Hb) was 146 g/L, platelet count (Plt) was 248 × 10^9^/L, and activated partial thromboplastin time (APPT) was 20.8 s (reference range: 24.3–36.0 s). Pharyngeal fiberoptic findings showed a small amount of venous hemorrhage from the posterior part of the left tonsil, and varicose veins were observed at the upper right soft palate (Figure 1). 

Considering the existence of varicose veins, the tendency of exacerbation of the hemorrhage was very low, as well as the risk of thrombosis, bypass therapy with factor VII was not performed. Tranexamic acid was administered, and spontaneous hemostasis was expected. Small amounts of melena were observed on the day after admission; however, there was no intraoral bleeding during the monitoring period. Approximately 200 mL of hematemesis was observed on day 3 of hospitalization. Hematologic tests showed a Plt of 156 × 10^9^/L and an APPT of 22.7 s, but Hb decreased to 83 g/L. Active hemorrhage from the posterior part of the left tonsil was observed on laryngeal fiberoptic findings (Figure 1), and urgent hemostasis ablation under general anesthesia was performed. The patient did not bleed postoperatively but was subsequently discharged on postoperative day 6. Varicose vessels were suspected to be caused by thrombus, and ultrasound examinations of the neck, heart, and abdomen were performed during hospitalization; however, no apparent thrombus was found. Furthermore, contrast-enhanced computed tomography, which was performed after discharge, did not suggest abnormal vessels or thrombus around the pharynx and tonsils. No further bleeding has been noted since discharge.

## 3. Discussion

This case revealed the following points. In hemophilia, severe traumatic hemorrhage can occur despite favorable emicizumab management. Moreover, in pediatric patients, physiologic tonsillar enlargement can be observed, and assessment of bleeding can be challenging. 

Emicizumab promotes coagulation reactions by bridging factors IXa and X. Due to this effect of emicizumab, APTT would be shortened and does not accurately reflect the capacity for coagulation. A cynomolgus monkey model suggested that emicizumab shortens APTT to the lower limit of normal or below [2,3,6], suggesting the same potential coagulative activity as that of 15% of factor VIII [7]. This is comparable with the mild form of hemophilia, and therefore, trauma or mucosal bleeding may be difficult to stop, despite a suppressed bleeding rate compared with conventional coagulation factor replacement therapy [3]. In this case, the use of bypassing agents was considered, but, as shown in the images, varicose veins were observed at the upper right soft palate during hospitalization. Under the use of emicizumab, thrombotic adverse events were reported in cases of prothrombin complex concentrate use but not recombinant activated factor VIIa use [3]. In addition, we performed ultrasound examination and contrast-enhanced computed tomography, which showed no thrombosis, varices, or other abnormalities. Therefore, the reasons for these varicose veins remain unknown. Owing to hematemesis and sudden Hb drop, emergency treatment was challenging, but cauterization was determined as desirable and was performed.

In pediatric patients, physiologic tonsillar enlargement because of repeated infections can be observed, making it difficult to perform detailed evaluations within the pharynx. Tonsillar hemorrhage is often observed following tonsil resection; however, the idiopathic type is most often because of increased blood flow associated with infections [8]. The patient was eight years old, and swelling was observed despite his age; therefore, monitoring was difficult, and the determination of the degree of hemorrhage was also problematic. Massive hemorrhaging in patients with hemophilia treated with emicizumab is unusual, and to the best of our knowledge, there have been no previous reports of tonsillar bleeding [3,9]. In this case, hemostasis was achieved with surgical intervention, but the use of bypassing agents is effective for hemorrhage caused by trauma during the use of emicizumab [10,11,12]. In patients with hemophilia, even during emicizumab induction, bypass therapy should be actively performed when the possibility of apparent thrombosis is not considered.

## 4. Conclusions

In patients with hemophilia A treated with emicizumab, normal APTT does not always accurately reflect the capacity for coagulation. Therefore, if the assessment of the source of bleeding is difficult in traumatic or mucosal bleeding, careful evaluation, including invasive tests such as fiberoptic testing, should be performed, and appropriate hemostasis should be attempted. Experience regarding emicizumab use in pediatric patients is limited, and evaluation of more patients with hemorrhage is warranted in the future.
